# Gait speed as a surrogate marker of change in cognitive function derived from multimodal lifestyle intervention: J‐MINT study

**DOI:** 10.1002/alz.086076

**Published:** 2025-01-09

**Authors:** Kosuke Fujita, Taiki Sugimoto, Nanae Matsumoto, Kazuaki Uchida, Yujiro Kuroda, Hidenori Arai, Takashi Sakurai, J‐MINT study group

**Affiliations:** ^1^ National Center for Geriatrics and Gerontology, Obu, Aichi Japan

## Abstract

**Background:**

The effectiveness of multimodal lifestyle interventions to prevent dementia is being validated. Since a relatively long period (∼2 years) is required for manifesting an impact on cognitive function, the exploration of an alternative marker that exhibits changes within a comparatively brief duration, thereby prognosticating future alterations in cognitive function, is needed. The decline in gait function is associated with cognitive impairment and is also a predictor of future cognitive decline. This study examined whether gait speed can be used as a surrogate marker for change in cognitive function derived from multimodal lifestyle interventions.

**Method:**

Data from a multi‐center, open‐label, randomized controlled trial in Japan (J‐MINT study) are utilized. Community‐dwelling older adults with mild cognitive impairment, aged 65 to 85 years, were enrolled in the J‐MINT study. The intervention group received 18 months of a multimodal lifestyle intervention (exercise, nutrition, cognitive stimulation, and vascular risk management), while the control group received only health information. Cognitive function was measured with a global composite score using eight neuropsychological tests. All variables were measured at baseline, 6, 12, and 18 months. Multiple regression analysis was performed for each of the allocation groups to investigate the association between the change in 18‐month cognitive function and the change in gait speed at 6 or 12 months from baseline.

**Result:**

Among 378 participants analyzed (baseline age, 74.2±4.8 years; 50% of male; gait speed of 1.25±0.24 m/s), 194 were the intervention group. Significant gait speed improvement was observed in the intervention group at 12 months when comparing the control groups (least square mean = 0.033). Multiple regression analysis revealed a significant association between changes in gait speed and cognitive function only in the intervention group (gait speed change in baseline to 6M, β = 0.452, p = 0.016; baseline to 12M, β = 0.387, p = 0.042).

**Conclusion:**

Short‐term changes in gait speed following multimodal interventions may serve as valuable surrogate markers, predicting of subsequent long‐term (approximately 18 months later) alterations in cognitive function. This suggests the potential of gait speed as an early indicator of intervention efficacy in preventing cognitive decline.

